# Susceptibility of Yellow Squash and Zucchini Cultivars to the Sweetpotato Whitefly, *Bemisia tabaci* Gennadius (MEAM1), in the Southeastern United States

**DOI:** 10.3390/insects15060429

**Published:** 2024-06-06

**Authors:** George N. Mbata, Yinping Li, Sanower Warsi, Alvin M. Simmons

**Affiliations:** 1 Entomology Research Laboratory, Agricultural Research Station, Fort Valley State University, 1005 State University Drive, Fort Valley, GA 31030, USA; yinpingli2011@gmail.com (Y.L.); sanower.warsi@fvsu.edu (S.W.); 2 United States Department of Agriculture—Agricultural Research Service (USDA ARS), U.S. Vegetable Laboratory, 2700 Savannah Highway, Charleston, SC 29414, USA; alvin.simmons@usda.gov

**Keywords:** vegetable, zucchini, yellow squash, whitefly, host plant resistance, yield

## Abstract

**Simple Summary:**

The sweetpotato whitefly, *Bemisia tabaci* Gennadius Middle East–Asia Minor 1 (MEAM1), is an economically important vegetable worldwide. This study evaluated the susceptibilities of yellow squash and zucchini cultivars to MEAM1 across three growing seasons in the southeastern United States: summer 2021, fall 2021, and fall 2022. Commercially available cultivars squash and zucchini were examined for resistance to MEAM1 infestations and yield performance. Weekly MEAM1 adult, egg, and nymph counts were conducted over 5 (fall 2022) or 6 (summer and fall 2021) weeks beginning after the third week of planting in each season. In general, MEAM1 adult populations were high at the first week of sampling but decreased in the subsequent weeks. The Zucchini cultivar ‘Black Beauty’ had the highest number of MEAM1 adults, and ‘Green Eclipse Zucchini’ had the lowest adult counts in summer 2021. For yellow squash, ‘Early Summer’ and ‘Amberpic 8455’ were identified as the cultivars that harbored the highest populations of adults in 2021, whereas ‘Golden Goose Hybrid’ harbored the least number of adult infestations in fall 2022. Generally, MEAM1 egg counts trailed adult peak populations. For yield performance, ‘Gourmet Gold Hybrid’, ‘Lioness’, ‘Fortune’, and ‘Golden Glory’ achieved the highest yields. These results provide valuable information for whitefly management in yellow squash and zucchini based on host plant resistance and yield.

**Abstract:**

The sweetpotato whitefly, *Bemisia tabaci* (Gennadius) Middle East–Asia Minor 1 (MEAM1), causes significant losses to vegetable crops directly by sap-feeding, inducing plant physiological disorders, and elevating the build-up of sooty mold, and indirectly by transmitting plant viruses. In this study, we evaluated the susceptibility of 20 yellow squash and zucchini (*Cucurbita pepo*) cultivars to MEAM1, across three growing seasons in the southeastern United States. Weekly sampling of the numbers of MEAM1 adults, nymphs, and eggs were conducted from the fourth week after seed sowing and across 6 weeks during the summer and fall of 2021 and five weeks during the fall of 2022. In general, adult whitefly populations were high during the first week of sampling but decreased as the seasons progressed. The zucchini cultivar ‘Black Beauty’ harbored the most adults, while ‘Green Eclipse Zucchini’ was the least attractive zucchini cultivar to the adults in fall 2022. For yellow squash, ‘Early Summer’ (summer 2021) and ‘Amberpic 8455’ (summer 2021 and fall 2022) were the cultivars with the highest adult populations, while ‘Lioness’ (summer 2021) and ‘Gourmet Gold Hybrid’ (fall 2022) harbored the lowest adult counts. The whitefly egg counts across both vegetables trailed those of adults and peaked in the second week of sampling. The counts of nymphs increased as the seasons progressed, but there was a decline after the second week during fall 2021. For the yellow squash cultivars, ‘Gourmet Gold Hybrid’, (summer 2021 and fall 2022), ‘Lioness’, and ‘Fortune’ (summer 2021) recorded the highest yields. For zucchini, ‘Golden Glory’ (summer 2021) was the top performer. These results provide valuable information for whitefly management in yellow squash and zucchini based on host plant susceptibility and yield.

## 1. Introduction

Cucurbits (squash and zucchini, *Cucurbita pepo* L.) are among the most important cultivated crops worldwide and are economically important [1]. The United States is a significant producer of cucurbits. In 2019, squash production reached 3.21 million kg, valued at USD 219.9 million, across 43,500 acres in the United States [2]. In the state of Georgia alone, yellow squash and zucchini cultivation contributed USD 63.5 million across 6391 acres in 2019 [3]. However, from 2000 to 2020, the production of cucurbits declined [4]. The total production of fresh squash dropped from 400 million kg in 2000 to 227 million kg in 2020, down 43% over 20 years [4]. One of the reasons for the declining production of squash is the sweetpotato whitefly, *Bemisia tabaci* Gennadius (Hemiptera: Aleyrodidae) Middle East–Asia Minor 1 (MEAM1) [4].

Studies have shown that whiteflies prefer cucurbits more than solanaceous plants [5,6,7]. Both adults and immatures feed on the sap, leading to chlorosis and necrosis spots, and honeydew excretion facilitates the growth of sooty mold [8,9,10,11]. Their feeding also causes silver leaf disorder and tarnished leaf appearance and fruit color, which reduce the crop’s marketability [12]. Along with direct losses due to a reduction in nutritional values, MEAM1 can indirectly impair the host plants by transmitting pathogenic viruses [1,4,10,11]. This sap-sucking pest can transmit over 300 plant viruses; therefore, it is known as a supervector [13]. In 2016, almost 70% of the squash grown in southeastern Georgia and north central Florida was infected with whitefly-transmitted *Cucurbit leaf crumble virus* (CuLCrV) [14,15]. It was estimated that whiteflies and whitefly-transmitted viruses led to yield reductions of 35% in 2017 and 15% in 2018 in Georgia [16,17]. These inherent characteristics of MEAM1 as a crop pest and a plant disease vector have made MEAM1 a harmful pest to a wide range of crops, including squash [18].

The management of MEAM1 populations is important for the successful production of squash crop in the southern United States. In conventional systems, growers currently rely on calendar sprays with chemical pesticides as the sole management technique against key pests of squash [19,20]. However, overuse of synthetic insecticides has led to various side effects, including insecticide resistance [21]. In addition, nymphal stages of whiteflies occur on the underside of the leaves, which are difficult to reach by spraying insecticides [15]. Chemical insecticides have also been reported to harm non-target insects, such as pollinators and natural enemies [22,23]. As cucurbits rely on pollinators for a good fruit set, the negative impact of insecticides on pollinator populations would drastically reduce cucurbit crop yield [24]. Therefore, alternative pest management strategies that are eco-friendly and efficient, such as resistant host plants, are needed for cucurbit cultivation [25]. Due to differences in food quality, morphological characteristics, and other host-dependent factors, the performance of MEAM1 may differ on plant cultivars [25,26,27,28]. Plant resistance is typically classified into three main categories: tolerance, antibiosis, and antixenosis [29,30]. Tolerance is the ability to endure damage caused by pests or diseases without experiencing significant reductions in yield or quality [31,32]. It may involve rapid regrowth, compensatory growth, or physiological adjustments [31,32]. Antixenosis is a genetic resistance of plant that involves changes in physical or chemical traits in plants that make them unattractive or unsuitable as a host [33,34,35]. Lastly, antibiosis deters pests from feeding, ovipositing, or colonizing the plant through the production of substances or morphological changes in plants that adversely affect the behavior of pests [36]. The importance of host plant resistance in integrated pest management has stimulated the study of categories of plant resistance as they relate to the interaction of different crops with MEAM1 [25,37,38,39,40]. For example, some common bean cultivars, such as IAC-Una and IAC-Eldorado, were documented as less susceptible to MEAM1 infestation [41]. A recent study by Li et al. [25] showed the ‘Jade’ snap bean cultivar had significantly fewer egg counts whereas ‘Gold Mine’, ‘Golden Rod’, ‘Long Tendergreen’, and ‘Royal Burgundy’ cultivars also had lower nymph counts. Therefore, the objectives of this study were to determine the susceptibility of 20 commercially available squash cultivars to MEAM1 and compare squash yield among these cultivars.

## 2. Materials and Methods

### 2.1. Experiment Site

The field experiments were conducted during 2021–2022 at the Fort Valley State University New Research Farm (32°31′11″ N, 83°52′2″ W) in Fort Valley, GA, USA. The climate of this region is humid subtropical [42], characterized by hot, humid summers and mild to cool winters. The soil of Fort Valley is characterized as ultisols (red clay soil) [43].

### 2.2. Experimental Design and Crop Management

MEAM1 population dynamics were evaluated in 10 commercially available yellow squash and 10 zucchini cultivars grown by Georgia vegetable growers (detailed in Table 1). Summer 2021 had 18 cultivars of squash (9 of yellow squash and 9 of zucchini). Fall 2021 and 2022 had 19 cultivars (10 cultivars of yellow squash and 9 of zucchini). We employed a randomized complete block design with three replications, using location as a blocking factor to account for environmental variations. Each of the three blocks contained 20 experimental plots, with each plot randomly assigned to each yellow squash or zucchini cultivar. Each plot comprised four rows, ensuring a uniform structure for observations across all cultivars.

Seeds were sown directly on 20 May 2021 in summer, 3 September in fall 2021, and 16 September in fall 2022. Planting was carried out at a depth of 2.5 cm. Rows had a length of 10.6 m and width of 0.91 m, with a spacing of 0.30 m between seeds within each row. Each variety, within a single block, consisted of four rows, totaling 36 plants per row. Irrigation, fertilization, and treatments with fungicides and herbicides were conducted following the standard protocol for Georgia. In the fall and summer 2021 and fall 2022 seasons, the experimental fields were treated with Command 3 ME herbicide (active ingredient: clomazone; FMC Corporation, Philadelphia, PA, USA) at 467.33–781.33 mL/ha. Insecticides were not applied in the experimental fields during any of the three seasons. During the three seasons, the soil was fertilized with N-P-K before sowing (nitrogen–phosphorus–potassium) (19-19-19) at 224 kg/ha. For the three seasons, the experimental fields were mainly irrigated by rainfall and watered overhead once using a farm irrigation sprinkler on the fourth week of sampling because of sparse rainfall.

### 2.3. MEAM1 Sampling

This study involved weekly monitoring of MEAM1 whitefly populations (eggs, nymphs, and adults), starting on the fourth week following sowing and continuing for six weeks (Table 2). To ensure accuracy and minimize variability, sampling was limited to the two central rows of each experimental plot, excluding the outer rows. Each plant within these rows was individually numbered for systematic sampling, with a random number selection method used for each sampling event. Selected plants were examined, with one leaf gently turned to count adult whiteflies before detachment. Two leaves from one plant’s upper, middle, and lower sections in total were randomly chosen and sampled using the above method, with one leaf from one plant. The detached leaves were carefully placed in labeled interlocking seal plastic bags and transported to the laboratory, where they were stored in a cool chamber (model PGC-9/2, Percival Scientific, Perry, IA, USA) at 4 °C. Subsequently, the eggs and nymphs on these leaves were counted using a Leica EZ4 W dissecting microscope (Leica Microsystems Inc. Buffalo Grove, IL, USA).

### 2.4. Squash Fruit Yield Evaluation

To evaluate the yield of squash and zucchini fruits, three healthy plants were randomly selected and marked by placing a small flag with the information on block, variety, and plant in each experimental plot (cultivar). If all the fruits (one or more than one) on each selected plant met the harvest standard, they were harvested. The harvest standard was that zucchini had to be about or exceed 20 cm in length, and yellow squash had to be about or exceed 20 cm in circumference. Zucchinis were identified as all green and yellow fruits, while all other yellow fruits were classified as squash. On each sampling date, the marked plants were located using the records, each plant was thoroughly checked, and the fruits were measured. Fruits that met the harvest criteria were removed, placed in a grocery bag, and weighed using a hanging fishing scale (Dr. Meter ES-PS01, Dr.meter, Columbus, OH, USA). The harvesting, weighing, and recording of fruits from each selected plant were carried out three times a week (Monday, Wednesday, and Friday), starting from the third week until the end of the experiment.

### 2.5. Statistical Analysis

Statistical analyses were performed using the software SAS ver 9.4 (SAS Institute, Cary, NC, USA) [44]. In our investigation focusing on the screening of yellow squash and zucchini cultivars for the relative incidence of MEAM1 populations, we employed a gamma distribution with a log link function to model the counts of MEAM1 adults, eggs, and nymphs. The linear predictor in the model included the fixed effects of years (2021 and 2022), treatment (20 cultivars), time (weeks 1 through 6), as well as two-way and three-way interactions. Random effects associated with the linear predictor included block in each year, which allowed for recognition of the experimental unit for the year. Random effects in the linear predictors also included experimental plot nested within block and year, and this allowed for recognition of experimental plot as the experimental unit for cultivar, as well as the unit of repeated measures over weeks.

For yield data, we compared yield differences among cultivars. The yield for each cultivar was calculated as the total weight of all the fruits harvested throughout the entire duration of the plant’s production period. These data were incorporated into the model using cultivar as a fixed factor and block as a random factor to analyze the differences.

Over-dispersion was evaluated using the maximum likelihood based on fit statistic Pearson chi-square/DF. In this analysis, the counts for MEAM1 adults, eggs, and nymphs derived from least squares mean (LSmean), which were adjusted means, ensured accurate group comparisons by accounting for covariates, thus providing a balanced representative data across treatments (cultivars). The final statistical model used for inferences was fitted using residual pseudo-likelihood. Degrees of freedom were approximated, and estimated standard errors were adjusted using Kenward–Roger’s procedure. The statistical model was fitted using the PROC GLIMMIX procedure of SAS implemented using Newton–Raphson with ridging as the optimization technique. Relevant pairwise comparisons were conducted using Tukey or Bonferroni adjustments to avoid inflation of type I error due to multiple comparisons among the treatments.

## 3. Results

### 3.1. Summer 2021

#### 3.1.1. MEAM1 Population Dynamics

##### Yellow Squash

**Figure 1:**
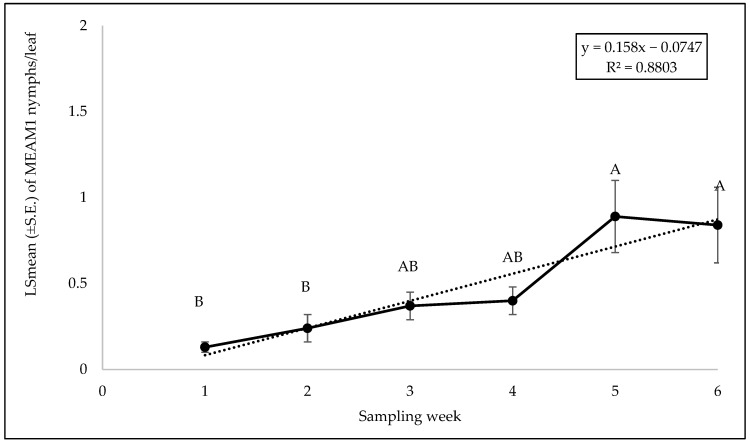
LSmean (±S.E.) of *Bemisia tabaci* Middle East–Asia Minor 1 (MEAM1) nymphs per leaf over six sampling weeks on yellow squash in the 2021 summer season. Different letters above the error bars indicate statistical significance (*p* < 0.05, Tukey’s test) among the sampling weeks.

The interaction between cultivars and sampling was not significant for MEAM1 adults (F = 1.32; df = 40, 105; *p* = 0.21), eggs (F = 0.46; df = 40, 105; *p* = 0.99), or nymphs (F = 1.33; df = 40, 105; *p* = 0.12). No significant differences were found in MEAM1 adult (F = 1.39; df = 8, 105; *p* = 0.21), egg (F = 0.91; df = 8, 105; *p* = 0.51), or nymph (F = 0.90; df = 8, 105; *p* = 0.51) counts among the cultivars. No significant difference was observed in MEAM1 adults (F = 1.39; df = 5, 105; *p* = 0.23) or eggs (F = 1.50; df = 5, 106; *p* = 0.19) across the sampling week. However, there was a significant difference in nymph counts over the six sampling weeks (F = 5.32; df = 5, 105; *p* = 0.0002). The MEAM1 nymph counts/leaf exhibited a stable trend initially but showed a significant increase by week 5, with a notable increase of about 5.46 from week 1 to week 6 (Figure 1). Approximately 88% (R^2^ = 0.880) of the variation in nymph counts was associated with the sampling period (Figure 1).

##### Zucchini

**Figure 2:**
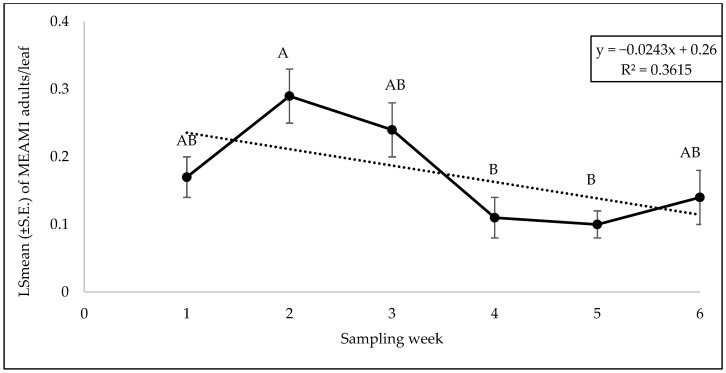
LSmean (±S.E.) of *Bemisia tabaci* Middle East–Asia Minor 1 (MEAM1) adults/leaf over six sampling weeks on zucchini in the 2021 summer season. Different letters above the error bars indicate statistical significance (*p* < 0.05, Tukey’s test) among the sampling weeks.

Like yellow squash, there was no significant interaction between cultivars and sampling weeks for adults (F = 0.99; df = 40, 106; *p* = 0.50), eggs (F = 1.50; df = 40, 106; *p* = 0.05), or nymphs (F = 0.97; df = 40, 105; *p* = 0.53). No significant difference was found in adult (F = 0.35; df = 8, 106; *p* = 0.94), egg (F = 0.85; df = 8, 106; *p* = 0.55), or nymph (F = 1.03; df = 8, 105; *p* = 0.41) counts among the cultivars. No significant difference was observed in MEAM1 egg (F = 0.51; df = 5, 106; *p* = 0.76) or nymph (F = 0.89; df = 5, 105; *p* = 0.49) counts across the sampling weeks. However, there were significant differences in MEAM1 adults (F = 3.73; df = 5, 106; *p* = 0.003) across the sampling week. The adult counts/leaf showed an initial increase, peaking at week 2, then a decline through to week 5, with a minor increase in week 6 (Figure 2). Approximately 36% (R^2^ = 0.361) of the variation in adult counts was associated with the sampling period (Figure 2).

#### 3.1.2. Yellow Squash and Zucchini Yield

**Figure 3:**
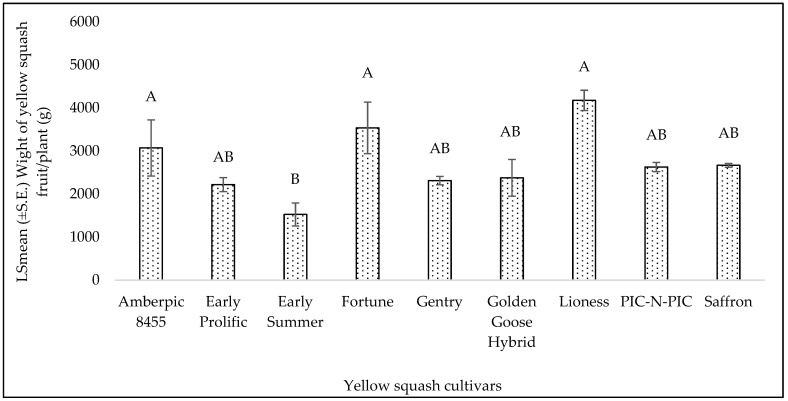
LSmean (±S.E.) of yellow squash fruit yield/plant (g) in summer 2021. Different letters above the bars indicate significant differences among cultivars (*p* < 0.05, Tukey’s test).

**Figure 4:**
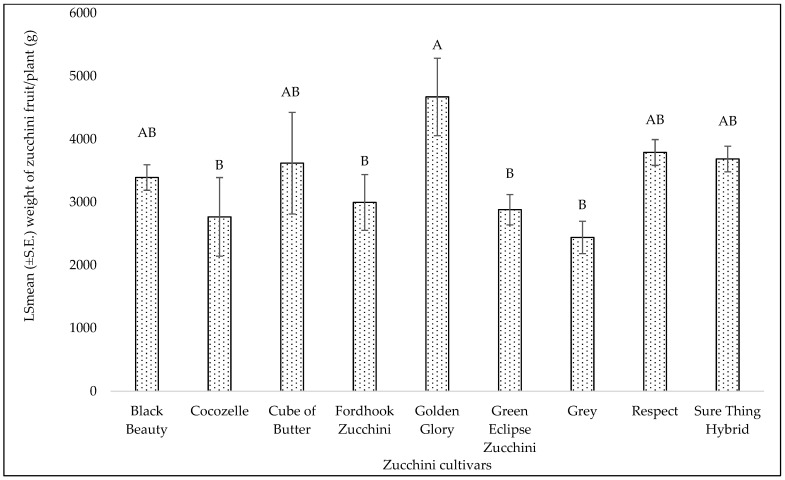
LSmean (±S.E.) of zucchini fruit yield/plant (g) in summer 2021. Different letters above the bars indicate significant differences among cultivars (*p* < 0.05, Tukey’s test).

The yield of yellow squash among different cultivars was significantly different (F = 4.82; df = 8, 16; *p* = 0.003) with ‘Lioness’, ‘Fortune’, and ‘Amberpic 8455’ yielding the highest at 4175.67, 3536, and 3070.83 g/plant, respectively (Figure 3). The lowest yield (1524.33 g/plant)-producing cultivar was ‘Early Summer’ in summer 2021 (Figure 3). The yellow squash yield ranged from 1524.33 to 4175.67 g/plant (Figure 3). Also, a significant difference in zucchini yield was observed (F = 2.90; df = 8, 16; *p* = 0.03). Cultivar ‘Golden Glory’ yielded the highest at 4670.78 g/plant (Figure 4). The fruit yield range among zucchini cultivars was from 2439.67 to 4670.78 g/plant.

#### 3.1.3. Correlations between MEAM1 Infestations and Climatic Factors

During summer 2021, the mean temperature had a strong negative correlation with the number of adult MEAM1 (r = −0.84). The mean relative humidity correlated moderately negatively with the adult population (r = −0.34), indicating that the number of adults slightly decreased as humidity increased. Rainfall did not correlate strongly with adult MEAM1 numbers (r = −0.03). The MEAM1 egg counts had a negative correlation with the mean temperature (r = −0.29) and a very weak negative correlation with the mean relative humidity (r = −0.06). Interestingly, there was a moderate positive correlation between egg counts and rainfall (r = 0.40). The correlation between egg counts and the number of adults was positive (r = 0.26). Nymph counts had weak negative correlation with mean temperature (r = −0.22) and showed a weak positive correlation with mean relative humidity (r = 0.22) and a moderate positive correlation with rainfall (r = 0.44).

### 3.2. Fall 2021

#### 3.2.1. MEAM1 Population Dynamics

##### Yellow Squash

**Figure 5:**
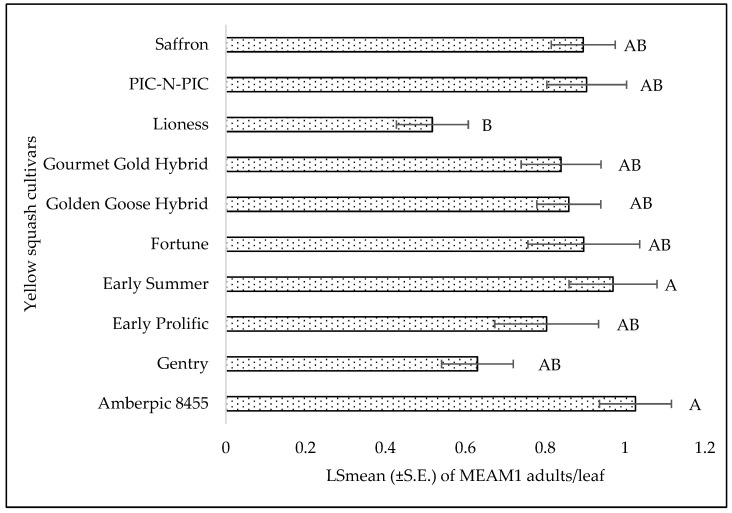
LSmean (±S.E.) number of *Bemisia tabaci* Middle East–Asia Minor 1 (MEAM1) adults/leaf on yellow squash cultivars during fall 2021. Different letters above the bars indicate significant difference (*p* < 0.05, Tukey’s test) among the cultivars.

**Figure 6:**
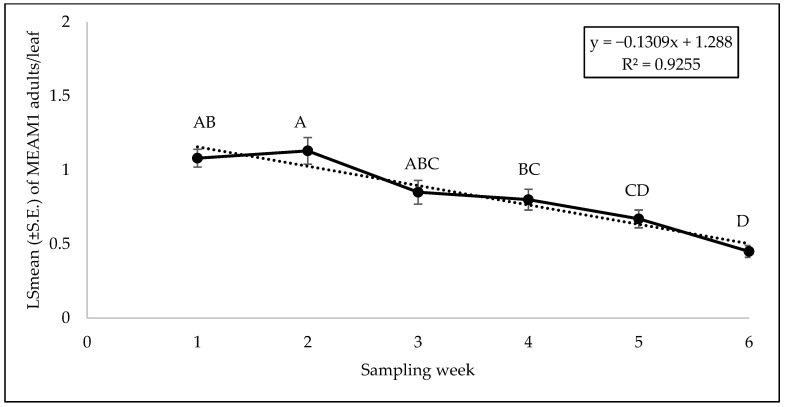
LSmean (±S.E.) number of *Bemisia tabaci* Middle East–Asia Minor 1 (MEAM1) adults/leaf on yellow squash cultivars during fall 2021 over six sampling weeks. Different letters above the bars indicate significant difference (*p* < 0.05, Tukey’s test) across the sampling weeks.

**Figure 7:**
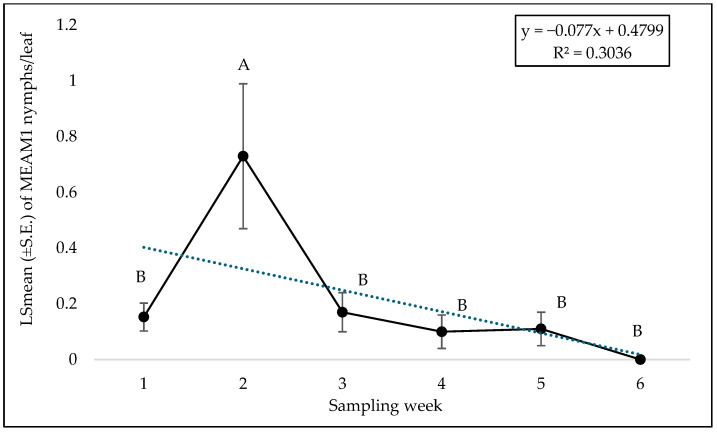
LSmean (±S.E.) number of *Bemisia tabaci* Middle East–Asia Minor 1 (MEAM1) nymphs/leaf on yellow squash cultivars during fall 2021 over six sampling weeks. Different letters above the bars indicate significant difference (*p* < 0.05, Tukey’s test) across the sampling weeks.

No significant interaction was found between cultivars and sampling weeks for adult (F = 0.46; df = 45, 118; *p* = 0.99), egg (F = 0.56; df = 45, 118; *p* = 0.98), or nymph (F = 0.79; df = 45, 118; *p* = 0.81) counts. A significant difference was found among squash cultivars for adult counts (F = 2.69; df = 9, 118; *p* = 0.0071). ‘Early Summer’ and ‘Amberpic 8455’ had the highest MEAM1 adult counts (Figure 5). In contrast, ‘Lioness’ had the lowest adult counts (Figure 5). However, there was no significant difference in egg (F = 0.70; df = 9, 118; *p* = 0.70) and nymph (F = 1.00; df = 9, 118; *p* = 0.44) counts among the yellow squash cultivars. A significant difference was found across sampling weeks for adults (F = 12.70; df = 5, 118; *p* < 0.0001). The mean counts of MEAM1 adults/leaf decreased about 58% from week 1 to week 6 (Figure 6). Approximately 92% (R^2^ = 0.92) of the variation in adult counts was associated with the sampling period (Figure 6). Nymph counts also differed significantly across the sampling week on yellow squash cultivars (F = 4.61; df = 5, 118; *p* = 0.0007). Mean counts of MEAM1 nymphs peaked in week 2 followed by a decline in numbers from the third week (Figure 7). Only 30% (R^2^ = 0.30) of the variation in nymph counts was associated with the sampling period (Figure 7). However, no significant difference was found for the eggs (F = 2.18; df = 5, 118; *p* = 0.60) across the sampling weeks.

##### Zucchini

**Figure 8:**
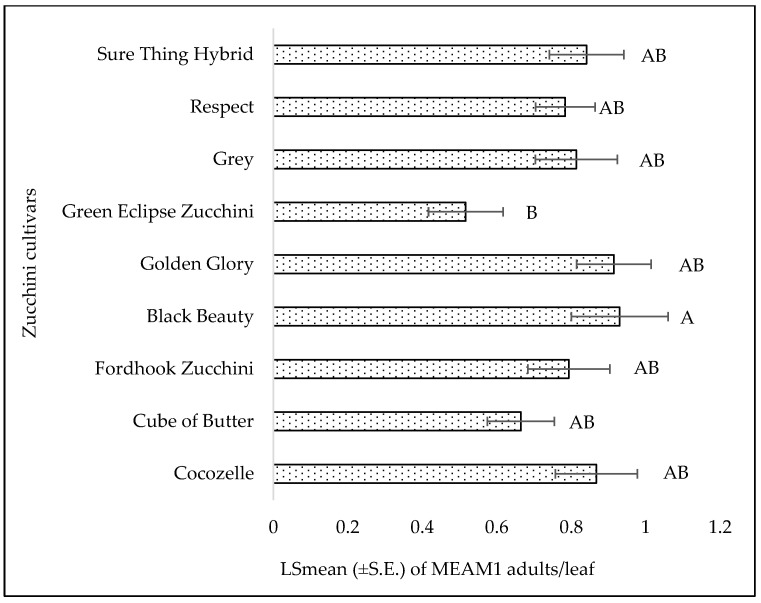
LSmean (±S.E.) number of *Bemisia tabaci* Middle East–Asia Minor 1 (MEAM1) adults/leaf on zucchini cultivars during fall 2021. Different letters above the bars indicate significant difference (*p* < 0.05, Tukey’s test) among the cultivars.

**Figure 9:**
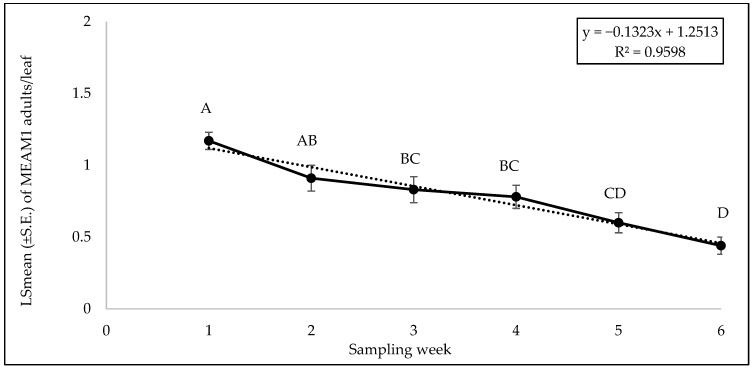
LSmean (±S.E.) number of *Bemisia tabaci* Middle East–Asia Minor 1 (MEAM1) adults/leaf over six sampling weeks during the 2021 fall season on zucchini cultivars. Different letters above the error bars indicate statistical significance (*p* < 0.05, Tukey’s test) among the sampling weeks.

**Figure 10:**
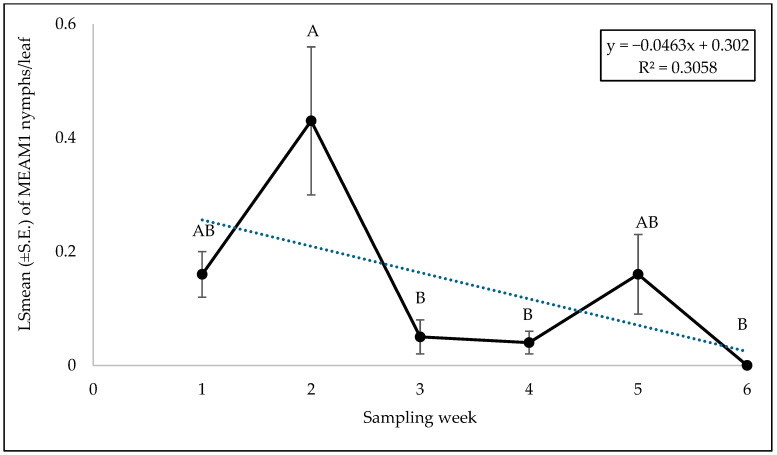
LSmean (±S.E.) number of *Bemisia tabaci* Middle East–Asia Minor 1 (MEAM1) nymphs/leaf over six sampling weeks during 2021 fall season on zucchini cultivars. Different letters above the error bars indicate statistical significance (*p* < 0.05, Tukey’s test) among the sampling weeks.

There was no significant interaction between the zucchini cultivars and sampling week for adults (F = 1.01; df = 40, 106; *p* = 0.46), eggs (F = 0.60; df = 40, 106; *p* = 0.96), or nymphs (F = 0.70; df = 40, 106; *p* = 0.90). Significant differences were observed for adults (F = 2.15; df = 8, 106; *p* = 0.03) among the zucchini cultivars. ‘Black Beauty’ had the highest MEAM1 adult counts, and ‘Green Eclipse Zucchini’ had the lowest adult counts (Figure 8). No significant differences were found in egg (F = 0.95; df = 8, 106; *p* = 0.48) or nymph (F = 0.75; df = 8, 106; *p* = 0.64) counts among the zucchini cultivars. The numbers of MEAM1 adults (F = 11.82; df = 5, 106; *p* < 0.0001) differed significantly across the sampling weeks. As the weeks progressed, the overall counts of MEAM1 adults on the zucchini plants decreased 62% from week 1 to week 6 (Figure 9). Approximately 95% (R^2^ = 0.95) variation in adult counts was associated with the sampling period, indicating a strong correlation between the sampling weeks and MEAM1 adult counts (Figure 9). The egg counts (F = 2.23; df = 5, 106; *p* = 0.05) were not significantly different over the six-week sampling period. The nymph (F = 4.85; df = 5, 106; *p* = 0.0005) counts significantly differed over the six-week sampling period. Like yellow squash, mean counts of MEAM1 nymphs peaked in week 2 followed by a decline in subsequent weeks (Figure 10). There was 30% (R^2^ = 0.305) variation in nymph counts associated with the sampling period (Figure 10).

#### 3.2.2. Yellow Squash and Zucchini Yield

The yellow squash (F = 1.02; df = 9, 18; *p* = 0.45) and zucchini (F = 2.49; df = 8, 16; *p* = 0.05) yields were not significantly different among the cultivars. The yield of yellow squash and zucchini fruit ranged from 992.78 to 2147.22 g/plant and 1035.00 to 3228.89 g/plant, respectively.

#### 3.2.3. Correlations between MEAM1 Infestations and Climatic Factors

During fall 2021, the correlation between mean temperatures and the adult MEAM1 counts was strong and positive (r = 0.93 at *p* < 0.01). Similarly, a strong positive correlation was found between mean relative humidity and adult MEAM1 numbers (r = 0.85 at *p* < 0.05). Rainfall showed a moderate positive correlation with adult numbers (r = 0.44). MEAM1 egg counts were positively correlated with mean temperature (r = 0.07), mean relative humidity (r = 0.31), and rainfall (r = 0.46). Nymph counts showed a moderate positive correlation with mean temperature (r = 0.54) and mean relative humidity (r = 0.22). However, nymph counts had a negative correlation with rainfall (r = −0.27).

### 3.3. Fall 2022

#### 3.3.1. MEAM1 Population Dynamics

##### Yellow Squash

**Figure 11:**
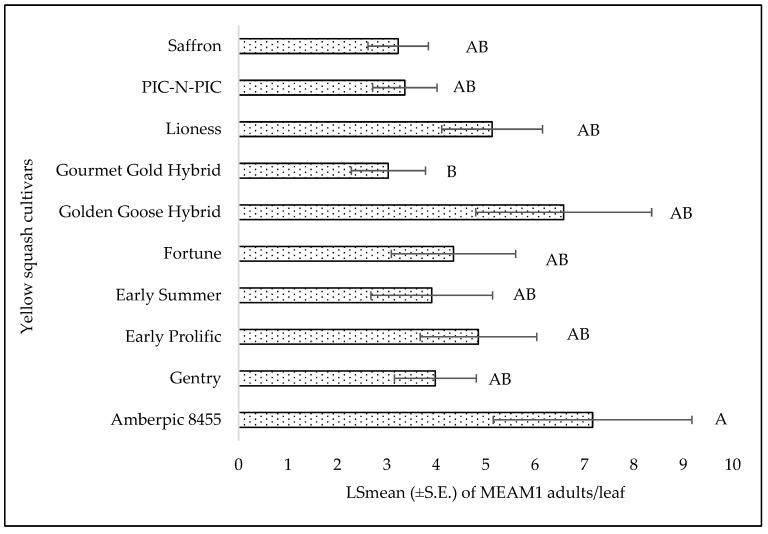
LSmean (±S.E.) number of *Bemisia tabaci* Middle East–Asia Minor 1 (MEAM1) adults/leaf on yellow squash cultivars in fall 2022. Different letters above the bars indicate significant difference (*p* < 0.05, Tukey’s test) among the cultivars.

**Figure 12:**
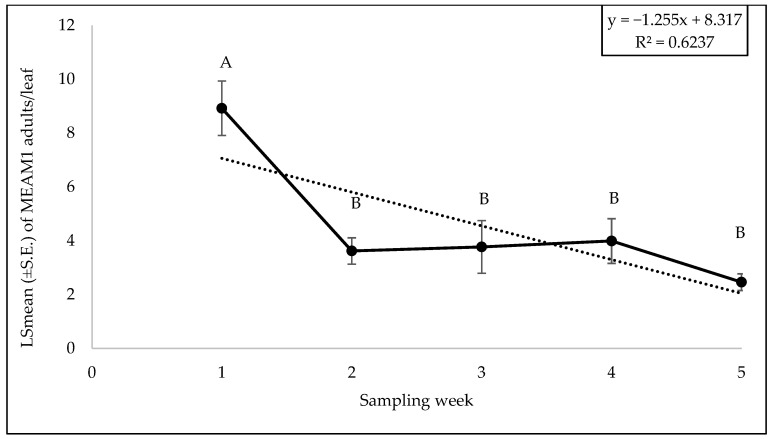
LSmean (±S.E.) number of *Bemisia tabaci* Middle East–Asia Minor 1 (MEAM1) adults/leaf over six sampling weeks in the 2022 Fall season on yellow squash cultivars. Different letters above the error bars indicate statistical significance (*p* < 0.05, Tukey’s test) among the sampling weeks.

**Figure 13:**
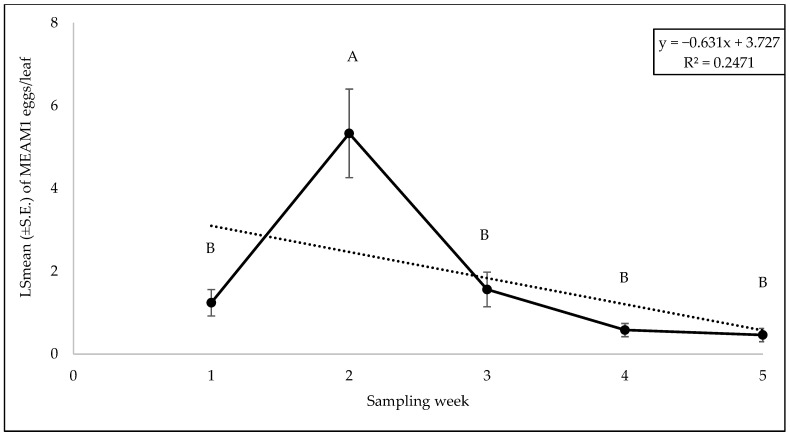
LSmean (±S.E.) number of *Bemisia tabaci* Middle East–Asia Minor 1 (MEAM1) eggs/leaf over six sampling weeks in the 2022 fall season on yellow squash cultivars. Different letters above the error bars indicate statistical significance (*p* < 0.05, Tukey’s test) among the sampling weeks.

**Figure 14:**
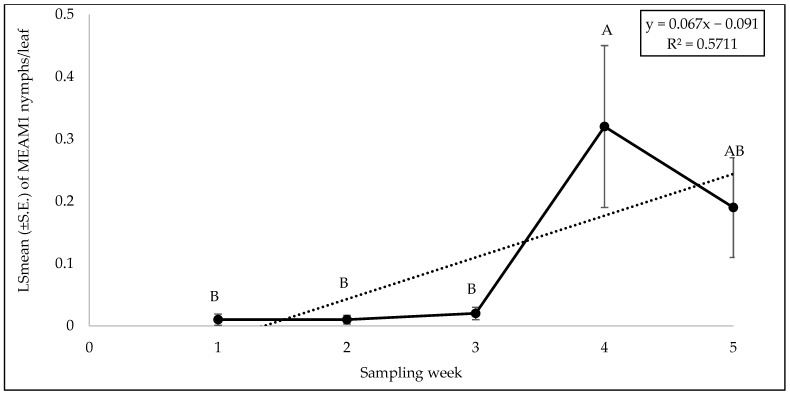
LSmean (±S.E.) number of *Bemisia tabaci* Middle East–Asia Minor 1 (MEAM1) nymphs/leaf over six sampling weeks in the 2022 fall season on yellow squash cultivars. Different letters above the error bars indicate statistical significance (*p* < 0.05, Tukey’s test) among the sampling weeks.

There was no significant interaction between cultivars and weeks with respect to adult counts (F = 0.62; df = 36, 98; *p* = 0.94), egg counts (F = 0.59; df = 36, 98; *p* = 0.96), and nymph counts (F = 1.02; df = 36, 98; *p* = 0.46). Among the different yellow squash cultivars, a significant difference was found for adults (F = 2.55; df = 9, 98; *p* = 0.01). The number of MEAM1 adults was the lowest on the cultivar ‘Golden Goose Hybrid’ and highest on ‘Amberpic 8455’ (Figure 11). However, no significant difference was found for egg (F = 0.66; df = 9, 98; *p* = 0.73) and nymph (F = 1.09; df = 9, 98; *p* = 0.37) counts among the cultivars. The numbers of MEAM1 adults differed significantly across the sampling week (F = 16.43; df = 4, 98; *p* < 0.0001). The maximum adult counts (8.80/leaf) were recorded on yellow squash cultivars in week 1 of fall 2022 (Figure 12) but subsequently declined over the weeks. About 62% (R^2^ = 0.62) variation in adult counts was associated with the sampling weeks. The MEAM1 egg (F = 13.06; df = 4, 98; *p* <0.0001) and nymph (F = 3.80; df = 4, 98; *p* = 0.0065) counts also differed significantly over the five-week sampling period. There was a noticeable peak in week 2, where the egg count increased significantly, followed by a decline in subsequent weeks (Figure 13). In week 1, the counts of MEAM1 nymphs were low. Significantly high numbers of nymphs were recorded in week 4 (Figure 14). Sampling weeks accounted for approximately 24% (R^2^ = 0.24) and 57% (R^2^ = 0.57) of the variation in egg and nymph numbers, respectively (Figure 13 and Figure 14).

##### Zucchini

**Figure 15:**
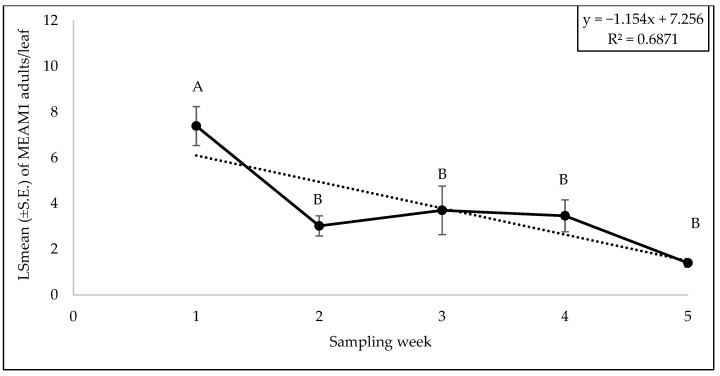
LSmean (±S.E.) number of *Bemisia tabaci* Middle East–Asia Minor 1 (MEAM1) adults/leaf over six sampling weeks in the 2022 fall season on zucchini cultivars. Different letters above the error bars indicate statistical significance (*p* < 0.05, Tukey’s test) among the sampling weeks.

**Figure 16:**
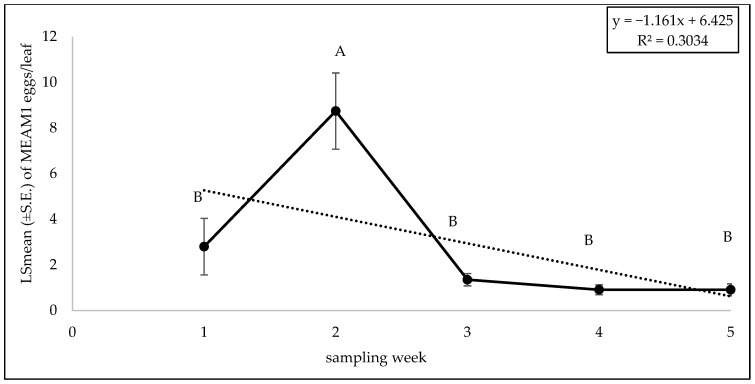
LSmean (±S.E.) number of *Bemisia tabaci* Middle East–Asia Minor 1 (MEAM1) eggs/leaf over six sampling weeks in the 2022 fall season on zucchini cultivars. Different letters above the error bars indicate statistical significance (*p* < 0.05, Tukey’s test) among the sampling weeks.

**Figure 17:**
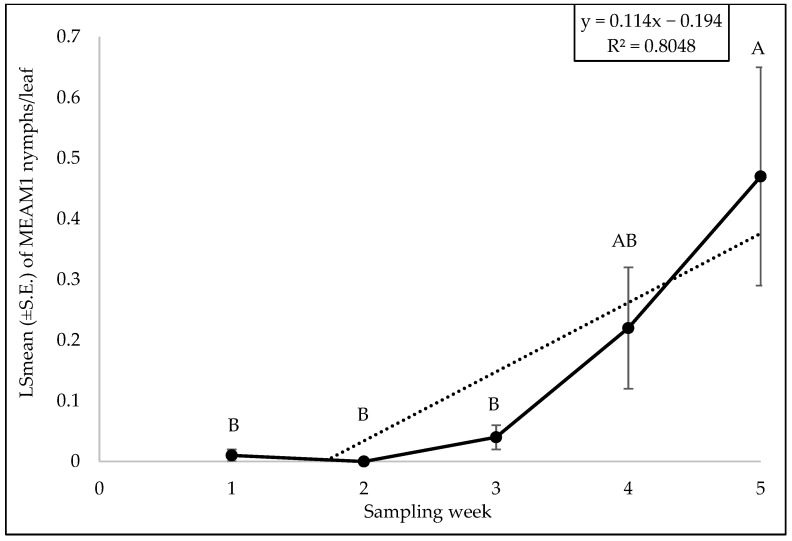
LSmean (±S.E.) number of *Bemisia tabaci* Middle East–Asia Minor 1 (MEAM1) nymphs/leaf over six sampling weeks in the 2022 fall season on zucchini cultivars. Different letters above the error bars indicate statistical significance (*p* < 0.05, Tukey’s test) among the sampling weeks.

The interaction between zucchini cultivars and sampling weeks did not significantly affect the adult (F = 0.81; df = 32, 88; *p* = 0.74), egg (F = 0.65; df = 32, 88; *p* = 0.91) or nymph counts (F = 0.87; df = 32, 88; *p* = 0.66). No significant difference was found for adult (F = 0.96; df = 8, 88; *p* = 0.46), egg (F = 0.35; df = 8, 88; *p* = 0.94), and nymph (F = 1.40; df = 8, 88; *p* = 0.20) counts among the different zucchini cultivars. The numbers of MEAM1 adults (F = 12.59; df = 4, 88; *p* < 0.0001) on zucchini cultivars differed significantly across the sampling weeks. The maximum adult numbers (7.39/leaf) were recorded in week 1 (Figure 15). About 68% (R^2^ = 0.68) variation in adult counts was associated with the sampling period (Figure 15). A significant difference was found for eggs for sampling weeks (F = 11.09; df = 4, 88; *p* < 0.0001). The MEAM1 egg counts peaked in week 2 (8.74 eggs/leaf) but declined in subsequent weeks (Figure 16). The variability in egg counts related to the sampling week was approximately 30.3% (Figure 16). A significant difference was found in nymph counts across the sampling weeks (F = 4.69; df = 4, 88; *p* = 0.0018). The MEAM1 nymph counts increased as week progressed (Figure 17). Nymph counts in week 5 were 46 times higher than in week 1 (Figure 17). Additionally, around 80% of the variation in nymph counts (R^2^ = 0.80) was associated with the time of sampling (Figure 17).

#### 3.3.2. Yellow Squash and Zucchini Yield

**Figure 18:**
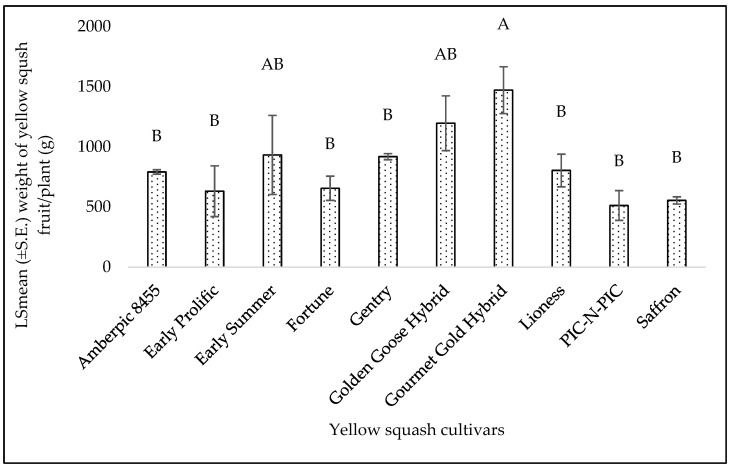
LSmean (±S.E.) of yellow squash fruit yield/plant (g) on cultivars in fall 2022. Different letters above the bars indicate significant differences among cultivars (*p* < 0.05, Tukey’s test).

The yields of yellow squash were significantly different among the cultivars (F = 3.38; df = 9, 18; *p* = 0.013). ‘Gourmet Gold Hybrid’ had the highest yield at 1470.89 g/plant, while ‘PIC-N-PIC’ has the lowest yield at 511.89 g/plant (Figure 18). However, the yield of zucchini did not differ significantly (F = 1.97; df = 8, 16; *p* = 0.11) among the cultivars. The range of yields found among the cultivars was from 511.89 to 1470.89 g/plant for yellow squash and from 1028.56 to 2306.67 g/plant for zucchini.

#### 3.3.3. Correlations between MEAM1 Infestations and Climatic Factors

During fall 2022, the correlation between MEAM1 adult numbers and the weather parameters was minimal to weak and non-significant. Adults had an almost zero correlation with mean temperatures (r = 0.006). A moderate negative correlation between adults and mean relative humidity (r = −0.52) was found. The relationship between adults and rainfall was also negative but weak (r = −0.16), indicating a slight decrease in adult counts with more rainfall. Egg populations also had a weak, but positive correlation with mean temperatures (r = 0.29) and mean relative humidity (r = 0.31). However, egg counts showed a moderate negative correlation with rainfall (r = −0.33). The MEAM1 nymph populations presented a moderate negative correlation with mean temperatures (r = −0.49) and a weak negative correlation with mean relative humidity (r = −0.18). However, there was a moderate positive correlation between nymph numbers and rainfall (r = 0.50).

## 4. Discussion

Host plant or cultivar preferences may affect MEAM1 distribution and dominance [18,36,45,46,47,48]. However, squash cultivars with resistance or tolerance to whitefly are not commercially available in the United States [49,50]. Although many studies have focused on screening squash cultivars for susceptibility to whitefly-transmitted viruses, these studies often did not prioritize the assessment of whitefly populations across different cultivars [51,52,53,54,55,56]. Some of these studies included adult MEAM1 counts among various genotypes, but only as a secondary consideration, possibly to investigate any correlation between levels of adult infestation and the incidence or severity of virus infections. For instance, Luckew et al. [49] screened 257 genotypes of *Cucurbita* for their resistance to whitefly-transmitted viruses and found notable differences in their attractiveness to or resistance against adult whitefly. However, that study did not provide information on whitefly eggs and nymph stages. The present study aligns with Luckew et al. [49] and supports significant differences in the tolerances of adult whiteflies to squash and zucchini cultivars. None of the evaluated yellow squash and zucchini cultivars showed complete resistance to MEAM1 infestations. For yellow squash, the ‘Lioness’ and ‘Golden Goose Hybrid’ cultivars exhibited the lowest infestation levels of MEAM1 (adults), while ‘Early Summer’ and ‘Amberpic 8455’ cultivars attracted the highest number of whitefly adults. In zucchini, ‘Green Eclipse Zucchini’ had the lowest MEAM1 (adults) infestation levels, while ‘Black Beauty’ had the highest number of MEAM1 adults. The variability in attractiveness among different cultivars can be attributed to a range of genetic factors that influence a plant’s physical and biochemical traits. Alves et al. [57,58] suggested that variation in secondary metabolites present in cultivars might be the main reason for differing levels of attractiveness to whitefly. Mrosso et al. [59] found a great variation in diterpenes, tetraterpenes, alkaloids, carotenoids, and fatty acid esters content among tomato cultivars, which influences the level of attractiveness of certain cultivars to whitefly. Similarly, Jaccard et al. [60] reported variations in cucurbitacin levels in different squash cultivars, which impact the feeding behavior of herbivores. On the other hand, physical attributes of plants, such as types of trichomes and their density, may affect the population dynamics of small sap-sucking insects [37,61,62]. Baldin et al. [63] reported that the high density of glandular trichomes in one of the tomato genotypes (LA-716) was the main cause of oviposition non-preference by the *B. tabaci* B biotype. This resistance is caused, in part, by the presence of 2-tridecanone found in the exudate of the four-lobed glandular trichomes [64].

Identifying susceptible or resistant squash cultivars based on the counts of MEAM1 (including adults and eggs) was of limited effectiveness because of the low populations of MEAM1 observed during the summer and fall of 2021. Furthermore, the summer 2021 was relatively cool and rainy in Fort Valley, Georgia [65]. However, the optimal conditions for whitefly development are temperatures between 20 and 33 °C, low relative humidity, and reduced rainfall [41,66,67]. Consequently, the weather conditions during summer 2021 may not have been conducive to the natural buildup of MEAM1 populations. As a result, MEAM1 populations remained low during the subsequent fall 2021 season.

There was a gradual decrease in the adult numbers following a peak in week 1 in the fall (2021 and 2022) seasons and week 2 in the summer season. The adult numbers were initially high but consistently declined with sampling weeks. This trend likely reflects an initial population of older adults that migrated from the wild to the field. These initial adults probably engaged in feeding and reproductive activities with the population gradually tapering off. It is also likely that whitefly preference for the early stages of the plants may partially explain this reduction in numbers over time. Whitefly prefers to feed and oviposit in the vegetative stage of plants [68] because, at this stage, the vegetative parts of plant accumulate energy and nutrients, which possibly shift from leaves to flowers, pods, and seeds as the plant matures [69]. Thus, MEAM1 adults will likely acquire the best nutrients for development during seedling stage of the squash and zucchini, which explains the highest number of adult MEAM1 in earlier weeks. Our findings align with previous studies [25,41,70] that found adult whitefly infestations were higher in the early stage of the plants.

The moderate rainfall in fall 2021 might have contributed to higher adult numbers, though not as much as mean temperatures or relative humidity.

In yellow squash and zucchini cultivars, the egg counts peaked in week 2 in fall 2022. Across the weeks, there was a noticeable decline in egg counts. The peak in egg counts in week 2 suggests synchronized oviposition by the initial influx of adults, followed by a gradual decline as nymphs hatched out. The shift in nutrient allocation in plants from leaves to reproductive parts as the season advanced also may influence whitefly oviposition behavior, aligning with the reduction in egg counts over time. The nymph numbers peaked in week 2, followed by a decline in fall 2021. It corresponds to relatively mild temperatures and high relative humidity. These conditions are conducive for rapid whitefly development from eggs to nymphs. The substantial rainfall during the first week of fall 2021 could have also affected plant vigor and, consequently, the suitability of the host plants for whitefly oviposition and nymph development. Conversely, fall 2022 exhibited a late peak in nymph numbers, with a noticeable increase observed from the third week onwards. This shift can be linked to the initially cooler and drier conditions at the start of the fall season, which may have delayed the development of whitefly populations or their migration to the host plants. The gradual warming trend and increasing humidity by the third week of fall 2022 may have created more favorable conditions for whitefly reproduction and development. This apparently resulted in the observed peak in nymph numbers in the later weeks.

Our results indicate that among the yellow squash cultivars, ‘Lioness’, ‘Fortune’, and ‘Amberpic 8455’ yielded the highest in summer 2021 and ‘Gourmet Gold Hybrid’ yielded the highest in fall 2022. Despite higher infestation levels, ‘Amberpic 8455’ maintained substantial yields. For zucchini cultivars, ‘Golden Glory’, was the top performer in summer 2021. Interestingly, while ‘Green Eclipse Zucchini’ exhibited tolerance to whitefly infestations, its yield was lower than other cultivars more susceptible to pests. This observation suggests a potential trade-off between pest resistance and yield, where the allocation of resources to defense mechanisms might limit plant growth potential [71]. It should also be noted that the comparison of squash cultivars regarding yield was focused only on evaluating how each cultivar responded to MEAM1 infestations and their susceptibility to these pests. This comparison did not consider the inherent productivity of each cultivar, which can vary due to genetic factors. Namely, some cultivars may naturally produce higher yields even with pest infestations. Despite this limitation, top-performing cultivars with lower susceptibility to whitefly were identified. Future research will consider both the impact of pest infestations and the inherent productivity of squash cultivars to provide a more comprehensive understanding of their yield potential and losses to whitefly.

## 5. Conclusions

Based on this study, the ‘Lioness’ and ‘Golden Goose Hybrid’ in yellow squash cultivars and ‘Green Eclipse Zucchini’ in zucchini cultivars were identified as least susceptible to MEAM1 infestations. Among the yellow squash, ‘Lioness’, ‘Fortune’, and ‘Amberpic 8455’ and in zucchini cultivars, ‘Golden Glory’ were identified as top yielding cultivars. These cultivars are noteworthy for consideration by local vegetable growers in the southern United States. This study also revealed a temporal dimension to these interactions, with variations in MEAM1 numbers across weeks, highlighting the influence of environmental conditions, life cycle, and plant growth stages on pest populations. The strategic cultivation of these specific cultivars could serve as a natural pest control method, potentially reducing the reliance on chemical insecticides, mitigating insecticide resistance, and safeguarding the effectiveness of current pest management tools. This study’s findings underscore the necessity for adaptive pest management strategies that account for the seasonal variations in MEAM1 infestations, aligning agricultural practices with environmental conditions to optimize crop health and yield.

## Figures and Tables

**Table 1:** Yellow squash and zucchini cultivars used in the field experiments conducted during the summer 2021, fall 2021, and fall 2022 seasons.

Cucurbits	No.	Cultivar	Seed Vendor
Yellow squash	1	Early Summer	Burpee
	2	PIC-N-PIC	Burpee
	3	Saffron	Burpee
	4	Early Prolific	Burpee
	5	Lioness	Seedway
	6	Fortune	Seedway
	7	Amberpic 8455	Amazon
	8	Golden Goose Hybrid	Burpee
	9	Gourmet Gold Hybrid	Burpee
	10	Gentry	Seedway
Zucchini	11	Caserta	Amazon
	12	Grey	Amazon
	13	Cocozelle	Amazon
	14	Cube of Butter	Amazon
	15	Respect	Seedway
	16	Golden Glory	Seedway
	17	Fordhook Zucchini	Burpee
	18	Sure Thing Hybrid	Burpee
	19	Green Eclipse Zucchini	Seedway
	20	Black Beauty	Burpee

Note: Summer 2021 did not have ‘Gourmet Gold Hybrid’ and ‘Caserta’ cultivars. Fall 2021 and 2022 did not have ‘Caserta’ cultivar. These cultivars did not grow.

**Table 2:** Climatic data (mean ± S.E.) [temperature, T. (°C); relative humidity, RH. (%); and rainfall (mm)] in the weeks of sampling *Bemisia tabaci* Middle East–Asia Minor 1 on squash in the summer 2021, fall 2021, and fall 2022 seasons in Fort Valley, GA, USA.

	Mean ± S.E.			
	Sampling Week (Dates)	T (°C)	RH (%)	Rainfall (mm)
Summer 2021	1 (21–27 June)	24.48 ±0.34	77.67 ± 2.43	0.00 ± 0.00
	2 (28 June–4 July)	24.46 ± 0.30	83.68 ± 2.90	0.30 ± 0.15
	3 (5–11 July)	24.39 ± 0.44	84.86 ± 2.36	0.36 ± 0.16
	4 (12–18 July)	25.38 ± 0.39	82.13 ± 1.58	0.15 ± 0.15
	5 (19–25 July)	25.26 ± 0.34	87.94 ± 1.56	0.24 ± 0.12
	6 (26 July–1 August)	27.26 ± 0.27	81.29 ± 0.70	0.17 ± 0.11
Fall 2021	1 (4–10 October)	21.67 ± 0.41	91.80 ± 2.01	7.98 ± 4.63
	2 (11–17 October)	19.81 ± 0.98	79.65 ± 3.77	0.04 ± 0.04
	3 (18–24 October)	16.91 ± 0.88	75.63 ± 1.89	0.07 ± 0.05
	4 (25–31 October)	15.26 ± 1.04	79.78 ± 2.23	4.68 ± 4.68
	5 (1–7 November)	11.82 ± 0.92	70.28 ± 4.06	0.07 ± 0.05
	6 (8–14 November)	12.72 ± 1.26	71.58 ± 2.85	1.67 ± 1.59
Fall 2022	1 (17–23 October)	12.09 ± 1.46	57.8 ± 3.26	0.0 ± 0.00
	2 (24–30 October)	16.79 ± 0.41	75.16 ± 3.91	0.36 ± 0.21
	3 (31 October–6 November)	19.12 ± 0.82	77.90 ± 2.66	0.40 ± 0.40
	4 (7–13 November)	17.28 ± 1.91	72.99 ± 6.53	6.50 ± 4.18
	5 (14–20 November)	7.54 ± 0.89	62.85 ± 5.01	0.33 ± 0.33

## Data Availability

The data presented in this study are available in this article.
